# Related Biological Research in the Interface between Bone Cement and Bone after Percutaneous Vertebroplasty

**DOI:** 10.1155/2013/109784

**Published:** 2013-10-03

**Authors:** ZhenMing Hu, Gang Zhao, LiJun Wang, Bo Pu, Jie Hao, HanChang Lao, XiaoJun Zhang, Qiang Gan, Wei Jiang

**Affiliations:** ^1^Department of Spine Surgery, The First Affiliated Hospital of Chongqing Medical University, 1 Youyi Road, Chongqing 400016, China; ^2^Department of Orthopaedic Surgery, The Second Affiliated Hospital of Kunming Medical College, 1 Ma Yuan, Kunming, Yunnan 650101, China; ^3^Department of Orthopaedic Surgery, Dazu District Hospital of Chongqing, 138 West Longgang Road, Dazu District, Chongqing 402360, China

## Abstract

Percutaneous vertebroplasty (PVP) is widely used in the treatment of painful osteoporotic vertebral compression fractures with the injection of PMMA cement, and the controversy for PMMA damage to the osteoporotic bone tissue and to affect the fractures repairing never stops. 72 old female rabbits, each age 3.0~3.5 y, rabbits were assigned randomly to two groups of thirty-six each; PMMA cement were injected into vertebral body in rabbits via mimic PVP, sacrificed at 1 h, 24 h, 3 d, 7 d, 4 w, and 12 w. The expression VEGF and collagen type I, the tissue response, and repair reaction in the interface between PMMA and bone tissue were observed dynamically with RT-PCR and western blot technique; the osteocalcin expression were studied by immunohistochemistry. Compared with the control group, the expression of collagen I increased at 1 hour and was higher from 24 h to 3 d. From 4 weeks to 12 weeks after injection of PMMA. The expression of VEGF decreased at 1 hour and 24 hours, significantly increased at 3 days, decreased once again at 7 days, then increased significantly at 4–12 weeks. The osteocalcin expression continued to increase during 4 to 12 week. PMMA would not cause local bone permanent necrosis, and interface injury repairing cycle could be prolonged in a vertebroplasty.

## 1. Introduction

Percutaneous vertebroplasty (PVP), a persistent developing procedure, is nowadays widely used in the treatment of painful osteoporotic vertebral compression fractures with the injection of polymethylmethacrylate (PMMA) cement, which is accepted most in clinical surgery contributing for its effect of immediate pain relief, biomechanical function reconstruction, and low price, whereas PMMA was also denounced of goodish bone compatibility and combination [1–4]. Nevertheless, research is still being conducted to develop better injectable bone augmentation materials and biodegradable or bioactive bone “pastes” [5, 6]. Most researchers believe that pain relief is achieved through mechanical support and stability provided by the bone cement. The semisolid mixture of PMMA, an acrylic cement from monomer polymerization reaction, and exothermal reaction contributes to the cytoxicity and mutation toxicity in periapical tissue [7, 8]. Meanwhile, since vertebroplasty was first used in the treatment of painful osteoporotic vertebral compression fractures, the controversy for PMMA damage to the osteoporotic bone tissue and to effect the fractures repairing never stops [9, 10]; the postoperative biological influence and variation of the involved vertebrae has been less reported so far. The investigation was incorporated in an animal study that was performed to observe the biological changes on PMMA and bone interface after rabbit vertebroplasty in the sight of morphology and molecular biology. Evaluated contents consist of expression of vascular endothelial growth factor (VEGF) and collagen type I of bone interface tissue by RT-PCR and Western Blot, the osteocalcin expression was studied by immunohistochemistry.

## 2. Material and Methods

### 2.1. Animal Randomization

Seventy-two old New Zealand female rabbits (provided by KunMing Medical College, China), each aged from 3.0 to 3.5 years, weighed between 3 and 4.2 kg, were used in this study. All the rabbits were demonstrated as osteoporosis by DXA (Lunar, USA). Preoperative frontal and lateral position X-ray film demonstrates that all these rabbits had normal lumbar vertebrae sequences and bone architectures. Rabbits were assigned randomly to two groups of thirty-six each. All groups were treated with the same surgical procedures and evaluations but were sacrificed at different postoperative times at 1 h, 24 h, 3 d, 7 d, 4 w, and 12 w. All animals received general anesthesia using 4% of pentobarbital sodium (30 mg/kg) followed by helix veinintravenous injection. Considering elimination of biological variance caused by different level of vertebrae, injection (powder and liquid mixed at 20 g : 5 mL) of modified PMMA (Tianjin Synthetic Material Research Institute, China) applied to randomly selected lumbar vertebrae in the experimental group. The control group received operation alone, without PMMA injection. 

### 2.2. Animal Model Preparation and Material Implantation

A skin incision was made in the median of the back at the level of vertebral L_7_ (top of hip); the subcutaneous tissue and masseter muscle were divided to expose the anterior half of L_4_, L_5_, and L_6_. Referred to clinical PVP surgical procedures [3, 4], an epidural needle is advanced into the vertebral body via a transpedicular or parapedicular approach with injection depth 4 ~ 6 mm. The pasty PMMA is slowly injected into the corresponding vertebral body at dosage of 0.5 ~ 0.6 mL through an epidural needle connecting to a 1 mL volume syringe. The injection and extraction were performed in-synchronism. All above procedures applied to 2 vertebrae (L_2_, L_4_) of each rabbit. Postoperatively, intramuscular injection of 1 million units penicillin per day was doneuntil the 7th day, and all the rabbits were fed and monitored by the experimental animal center of our university. Rabbits were without death both before and after operation.

### 2.3. X-Ray Inspection

Each group of animals, respectively, took lateral X-ray film to inspect the presence, and morphologic change of PMMA before the animals was sacrificed at their predetermined time.

### 2.4. Sample Preparation

Each group of animals was sacrificed at their predetermined time; the vertebraes (L_2_, L_4_) were dissected and removed out the PMMA and cut into blocks containing the interface between bone cement and bone tissue, part of them for total tissues RNA extraction and the others for histology.

### 2.5. RT-PCR

Total RNA extraction of tissues [11, 12] TRIzol method (Invitrogen Company) was used to extract RNA out for agarose gel electrophoresis, detecting the completeness of RNA. RNA was subpackaged in store at −70°C (RT-PCR equipment, Bio-Rad).

Primer sequences are used in the experiment as follows:

 VEGF-L 5′-GAC ATC TTC CAG GAG TAC CC-3′ 157 bp
 VEGF-R 5′-TGA GGT TTG ATC CGC ATG AT-3′
 actin-L TGG CTC TAA CAG TCC GCC TAG 295 bp.

### 2.6. Western Blot

Total protein extraction was manipulated on ice and preserved at −70°C following the instruction of BCA-100 protein quantitation kit (Shanghai Biocolor BioScience & Technology Company).

The first antibody for 18 h was incubated under 4°C after diluted to 1 : 100 (VEGF) and 1 : 1000 (collagen), subsequently went through TBST rinshing for 4 × 10 min. The second antibody was put under ambient temperature for 1 h and TBST rinshing for 4 × 15 min. The PVDF film which was prior incubated in ECL reagent for 5 min was placed into the chromogenic film cassette. Finally in dark room carried out the exposure and developing with X-ray film according to an optimal fixing time.

### 2.7. Immunohistochemistry

The immunohistochemical procedure has been described previously reports [13], the avitin-biotin-complex method was used with ABC Peroxidase Staining Kit. The sections were incubated with rabbit anti-rabbit osteocalcin polyclonal antibodies; the negative controls were the four different grafted maxillary sinuses, where normal mouse IgG was used instead of primary antibodies for staining.

### 2.8. The Result Analysis

RT-PCR result computation: firstly, sample Ct-intraparameter Ct = ΔCt, to compute ΔΔCt we set a sample ΔCt (the control group as usual) as reference. Computing method was ΔCt (sample) −  ΔCt (reference). Outcome illustrated the mathematics relation between samples was 2 − (−ΔΔCt)th power.

Protein relative amount computation: place the X-ray film into gel Image Processing System (Tanon) to detect intensity and area of the target band. Relative amount = intensity × area.

### 2.9. Statistical Treatment

The data were represented in the form of mean ± standard deviation (X ± S) and analyzed utilizing the one-way ANOVA. The means of sample from both groups were compared using *t*-test. All statistical analyses were done by SPSS 13.0.

## 3. Results

### 3.1. General Observation after Spliting the Samples

Bone trabecula was full of PMMA cement that is tightly connecting to bone tissues and well distributed. PMMA was covered with considerable soft tissues and a few new born bone tissues in 12 w. In the control, soft tissues coverage and bone defect about 0.5 cm were visible. The bone defect whose volume kept invariableness was filled with hematoma and some granulation tissues but without new osteogenesis. 

### 3.2. X-Ray Investigation

All injected cement did not appear of any defluxion, crack, or loosening. The boundary between PMMA and bone became indistinct with new osteogenesis in subgroup 12 w.

### 3.3. Histomorphological Observation

**Figure 1:**
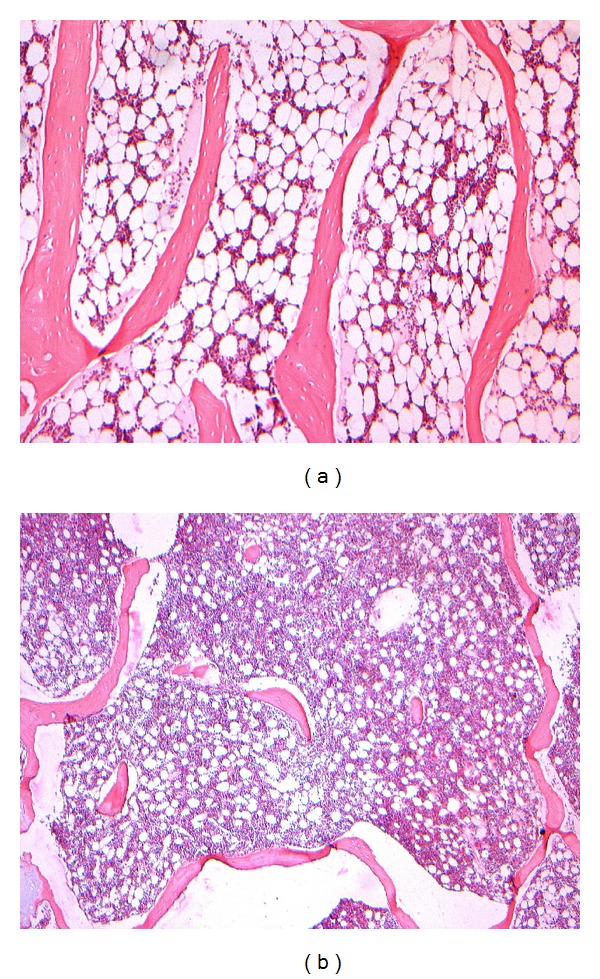
(a) Normal bone trabecula of vertebral in control, HE ×50. (b) Bone trabecula apparently reduced with inflammatory cells infiltration after PMMA injected 24 h, HE ×50.

**Figure 2:**
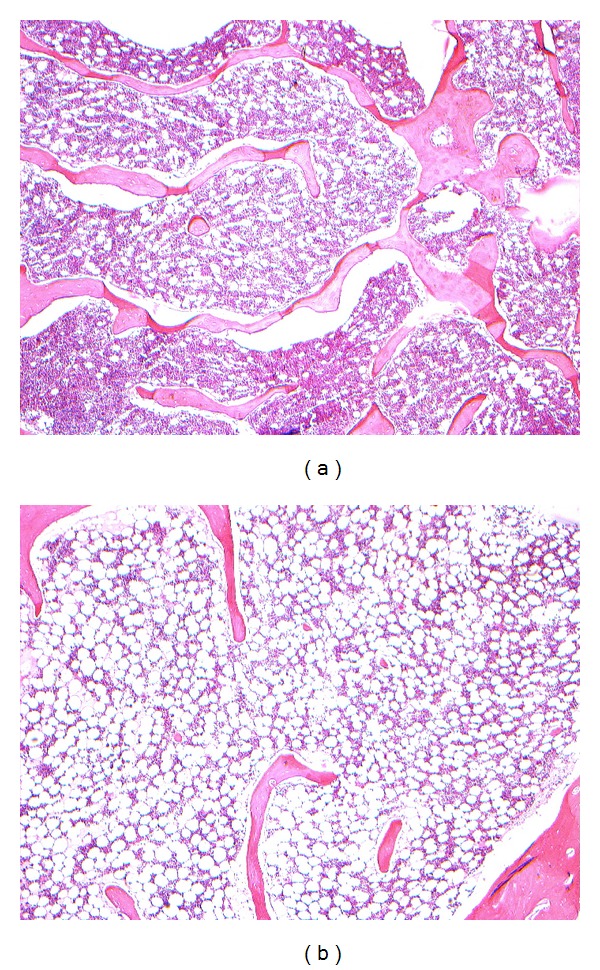
(a) Inflammatory cells infiltration after PMMA injected 3 d, HE ×50. (b) Inflammatory reaction lightened after PMMA injected 7 d, HE ×50.

**Figure 3:**
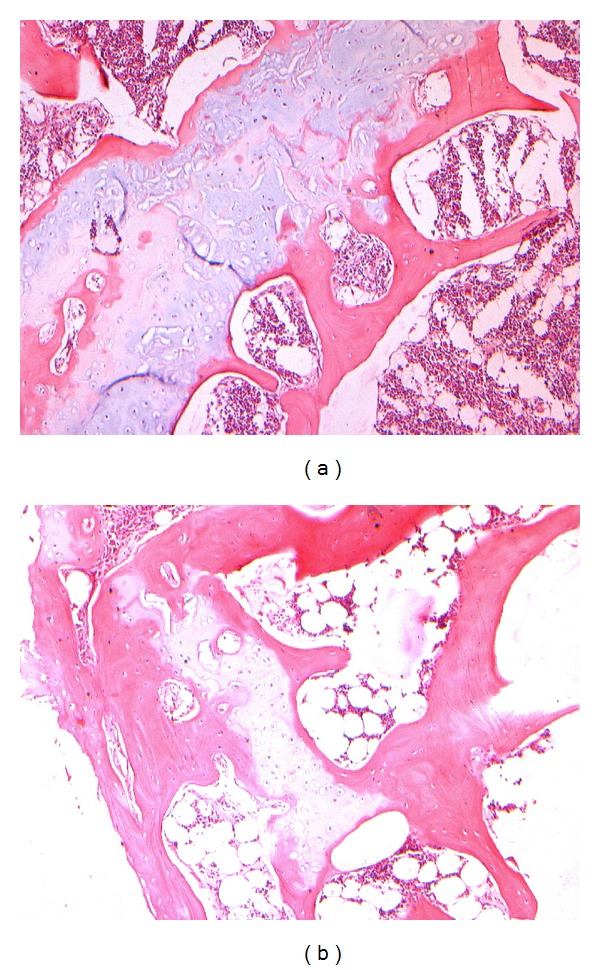
(a) Large quantity of chondrocytes growing in cluster after PMMA injected 4 w, HE ×100. (b) Chondrocytes in cluster without conspicuous inflammation after PMMA injected 4 w, HE ×100.

Compared with that of control, the samples were tightly combined with PMMA cement. The interface between bone tissues and PMMA was infiltrated with inflammatory cells and fibrous tissues. Inflammatory cell infiltration was obvious in 24 h, developed to a peak in 3 d, alleviated comparatively in 7 d. Chondrocytes were found growing in cluster and differentiating to woven bone at the interface without conspicuous inflammatory cells in 4 w; massive lamellar bone formed and occasional haematopoietic bone marrow could be observed without any inflammation in 12 w (Figures 1, 2, and 3).

### 3.4. VEGF RT-PCR Amplification and Protein Expression

VEGF was amplified to the target band of 157 bp molecular weight, taking actin as intraparameter. It confirmed the target band on coincidence to the gene fragment in the literature according to intraparameter.

### 3.5. VEGF RT-PCR Amplification Result

**Figure 4:**
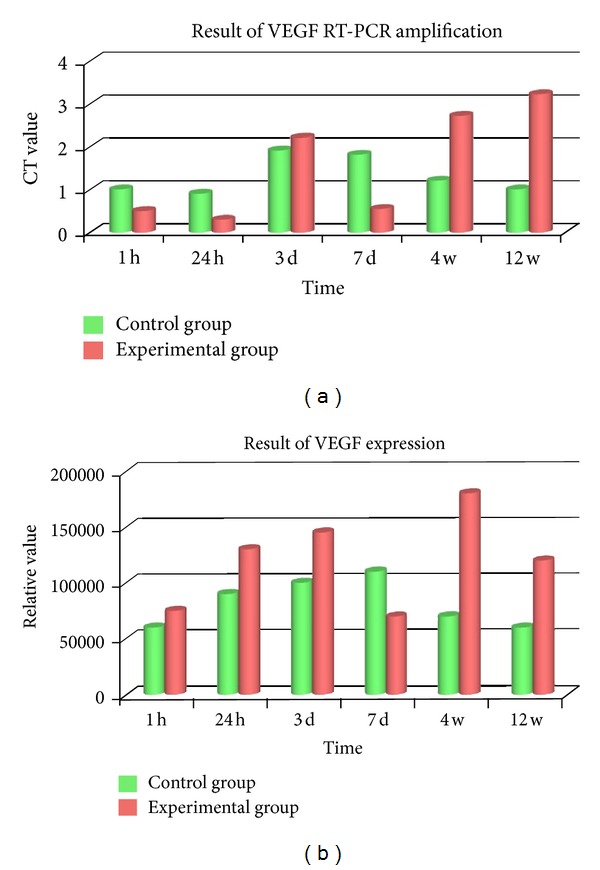
(a) VEGF mRNA expression was lower in 1 h and 24 h (*P* < 0.05), whereas the expression was highly increased in 3 d but decreased in 7 d. Then, it experienced a persistent augment from 4 w to 12 w (*P* < 0.001). (b) VEGF protein expression was increased in 1 h, 24 h, and 3 d; also it was decreased in 7 d. After that the expression was transparently elevated and higher than that of control (*P* < 0.05).

The VEGF mRNA expression was lower (*P* < 0.05) than that of control at 1 h and 24 h, whereas the expression was highly increased at 3 d. It was decreased and lower than the control again (*P* < 0.05) in 7 d. Then, it experienced a persistent augment from 4 w to 12 w; the difference was significant compared to the 7 d and control (*P* < 0.001) (Figure 4(a)). 

### 3.6. Detection Result of VEGF Protein

VEGF protein expression was increased a little at 1 h and 24 h. It was increased a lot in 3 d while decreased in 7 d. After that the expression was transparently elevated and higher than that of control  (*P* < 0.05) (Figure 4(b)).

### 3.7. Collagen Type I RT-PCR Amplification and Protein Expression

**Figure 5:**
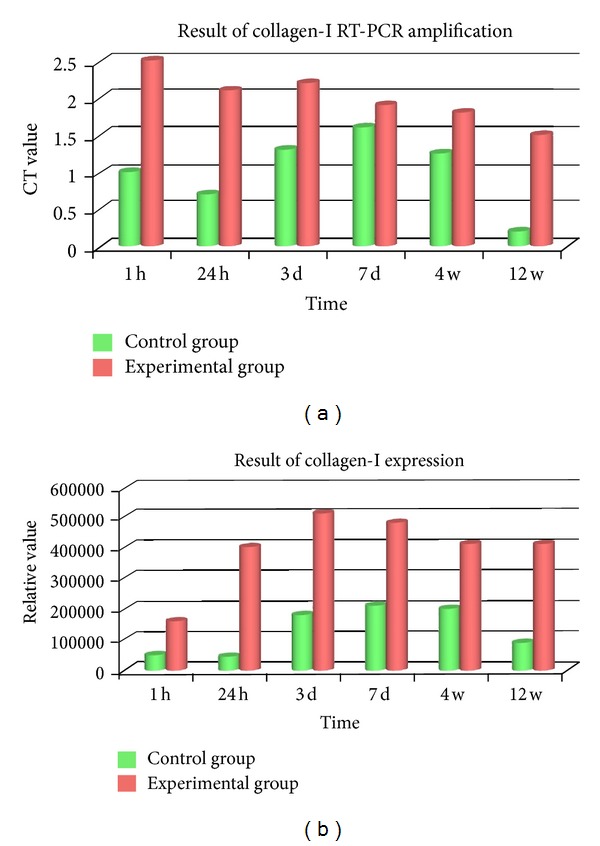
(a) Collagen type I mRNA expression was increased in 1 h. A persistent high level was formed in postoperative 24 h to 3 d. Despite being slightly descended in 7 d, it kept a comparative high expression from 4 w to 12 w which was remarkably higher than that of control (*P* < 0.01). (b) A high protein expression of Collagen type I was detected in 3 d, 7 d, and 4 w. The expression of 12 w descended considerably compared to 4 w with significant difference (*P* < 0.01).

Collagen type I mRNA expression was increased from 1 h. A persistent high level formed from postoperative 24 h to 3 d. Despite of being slightly descended in 7 d, it kept a comparative high expression from 4 w to 12 w which was remarkably higher than that of control (*P* < 0.01) (Figure 5(a)). High protein expression of Collagen type I was detected in 3 d, 7 d, and 4 w. The expression was descended at 12 w considerably compared to 4 w (*P* < 0.01) (Figure 5(b)). 

### 3.8. Immunohistochemistry

**Figure 6:**
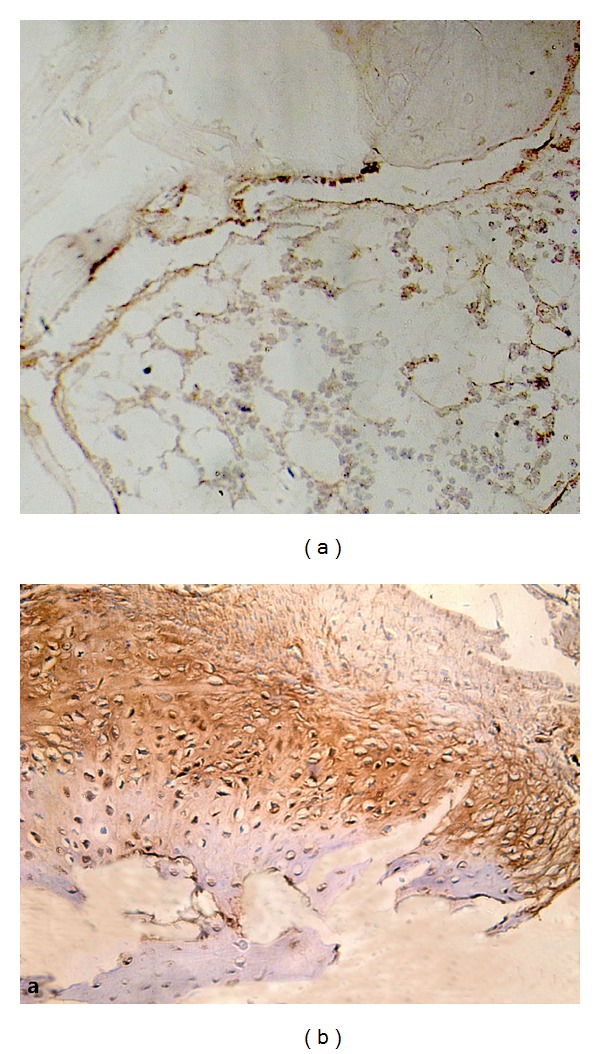
(a) OC expression slightly increased in PMMA-bone interface in 1 week, 20 × 10SP + HE. (b) OC expression in PMMA-bone interface in 4 weeks, when chondrocytes were found to be growing in cluster and differentiating to woven bone, 20 × 10SP + HE.

Compared with normal bone tissue, after 1 h, 24 h, and 3 d, the OC expression had no significant increase in both groups (*P* < 0.05). At 7 days, the OC expression of control group increased slightly, the PMMA group had no significant change (*P* > 0.05). At 4 weeks, the OC expression increased in both groups, but the control group was more significant than the PMMA group (*P* > 0.05). The OC expression continued to increase during 4 to 12 weeks in experimental group. It is slightly decreased in the control group (*P* > 0.05) (Figures 6(a) and 6(b)). 

## 4. Discussion

Several inherent advantages to PMMA include familiarity for orthopedic surgeons, ease of handling, good biomechanical strength and stiffness, and cost effectiveness [7–9, 14]. Several disadvantages, include no biologic potential to remodel or integrate into the surrounding bone, no direct bone apposition, excessive inherent stiffness, high polymerization temperature, and potential monomer toxicity [2, 3, 7, 10]. Although good clinical results have been reported in several series of both vertebroplasty and kyphoplasty procedures [15–21], it is still unclear whether some component of the pain relief is secondary to the mechanical stabilization, chemical toxicity, or thermal necrosis of surrounding tissues and nerve ends; in addition, the other surgery-related factors would affect the inflammatory progress. The concern regarding thermal bone necrosis is still theoretical, as to date, there has been no obvious evidence to support this [17, 22, 23]. In a baboon vertebral augmentation study, there were a few necrotic segment of bone present in both the vertebroplasty and kyphoplasty vertebrae. It was not, however, clear that the necrosis was caused by a PMMA polymerization process [22].

Lieberman et al. identified particles consistent with cement and/or barium sulfate in vascular spaces in human vertebrae obtained from surgical excision and autopsy cases [24, 25]. Our investigation of the histology and interfacial between cement and bone, PMMA induced somewhat inflammatory reaction, which subsided after 1 week postoperatively, totally disappeared at 4 weeks post-PVP. In which showed some component of the chemical toxicity, or thermal necrosis of interface tissues, the concern regarding thermal bone necrosis is still temporarily damage. Early research demonstrated that toxicity effect of PMMA induced osteoblast necrosis through peroxidation caused by hydroxy radical release. Free radical of PMMA monomer induces macrophages to release arachidonic acid in the interface of bone and cement [5–7]. Further, local tissues produce more lactate dehydrogenase resulting in granuloma like inflammation that aggravates tissue injury. Lately through improving compounding, PMMA has been proved high safety as the toxicity effect decreases [2]. In our investigation, we found that even though PMMA cement injected to stabilize or fix pathological fractures after vertebral augmentation, the painful osteoporotic compression fractures vertebral continued a healing and repairing processes [26].

The result of our research on VEGF illustrated in the 1 h and 24 h was lower than the corresponding of control (*P* < 0.05), whereas the expression of 3 d was highly increased; it was lower decreased at 7 d (*P* < 0.05). Then, it experienced a persistent augment from 4 w to 12 w. Descent of 24 h might ascribe to the exothermic reaction of PMMA solidification and oxidizing reaction of free radical monomer. The above two factors inhibit cytoactivity of osteoclast, contributing to lower VEGF expression [15, 27]. The first peak at 3 d results from an intensive inflammatory reaction which does favor to VEGF expression through inflammatory cells like macrophage and fibroblast; VEGF expression weakened along with extinction of inflammatory at 7 d. Accompanied with oxygen free radical reduction, repair of exothermic reaction injury, recovery of cellular microenvironment, and normalization of osteoblastic cytoactive, another high level of VEGF expression appeared. After osseous tissue repair is completed in 12 w, the VEGF expression was released mainly by osteoblasts [27, 28]. 

Meanwhile, in our studies, collagen type I at 1 h was multiple amplified, and a persistent high level formed from postoperative 24 h to 3 d. PMMA has the effect of promoting collagen information. Viateau et al. [29] discovered in an ovine study of padding bone defect that the membrane of PMMA concentrated BMP, bone stem cells, and other promotive osteoplastic components with vasiformation and collagen type I expression. Twa et al. [18] reported PMMA material implanted in vivo could activate keratocyte and collagen formation. Fibroblast is able to express collagen type I mRNA [30, 31]. The descendent at 7 d was probably caused by regression of inflammation. The high expression from 7 d to 12 w illustrated chondrocytes had been transforming to osteoblasts. HE staining revealed lamellar bone had been constructed 4 w postoperatively. Collagen type I is mainly produced by osteocytes, in our research it was expressed at a high level 12 w postoperatively which was distinct from conventional time (considerably decreased 8 w postoperatively). As same as VEGF and collagen type I, the OC showed high expression at the time point and delayed compared with control group. HE staining showed complete ossification accomplished at that time. However, the result of protein expression was similar to the literature.

In the process of fracture healing, collagen type I is the characteristic marker representing bone formation and molding. Its peak expression occurred between 3 w and 5 w after fracture which is the state from intracellular ossification of chondrocyte to bone information, being related to vasiformation. From the result of our study, PMMA cement does harm to bone trabecula at a certain degree in PVP. The major influence on osteoporotic fracture repair is time delay. Repair mechanism coincides with normal fracture healing, whereas the time is delayed for 4 weeks around. It is thus clear that, the PMMA injected in PVP influenced the function of local bone tissue and cell, but it did not induce irreversible damage.

In conclusion, interface injury caused by PVP injection of domestic advanced PMMA bone cement could be repaired and mineralized undergoing a process similar to normal fracture healing. However, the expression of VEGF, collagen type I, and OC delayed about 4 weeks. Bone cement would not cause local bone permanently necrosis; interface injury repairing cycle could be prolonged in a vertebroplasty. 

## Abbreviations

PVP: Percutaneous vertebroplasty
PMMA: Polymethylmethacrylate
VEGF: Vascular endothelial growth factor.
